# Manipulation of Barley Development and Flowering Time by Exogenous Application of Plant Growth Regulators

**DOI:** 10.3389/fpls.2021.694424

**Published:** 2022-01-03

**Authors:** Brendan M. Kupke, Matthew R. Tucker, Jason A. Able, Kenton D. Porker

**Affiliations:** ^1^School of Agriculture, Food & Wine, Waite Research Institute, The University of Adelaide, Urrbrae, SA, Australia; ^2^Agronomy Group, Crop Sciences Research Division, South Australian Research and Development Institute, Urrbrae, SA, Australia

**Keywords:** plant hormones, gibberellic acid, cytokinin, crop development, flowering

## Abstract

Matching flowering time to the optimal flowering period in Mediterranean cropping zones is pivotal to maximize yield. Aside from variety selection and sowing date, growers have limited options to alter development in season. Plant hormones and growth regulators are used in perennial horticultural systems to manipulate development and floral initiation. In this study, a range of plant hormonal products were tested to analyze their effects on barley (*Hordeum vulgare L*) development by exogenous spray applications. Plants were grown in controlled conditions under long and short photoperiods with different vernalization treatments. The gibberellin (GA) products demonstrated the greatest potential for altering development. The GA inhibitor trinexapac-ethyl was able to delay the time to flowering in genetically divergent barley cultivars by up to 200 degree days under controlled conditions. A similar delay in flowering could be achieved via application at both early (GS13) and late (GS33) stages, with higher rates delaying flowering further. Notably, trinexapac-ethyl was able to extend the duration of pre-anthesis phases of development. By contrast, GA3 was unable to accelerate development under extreme short (8 h) or long (16 h) day lengths. There was also little evidence that GA3 could reproducibly accelerate development under intermediate 10–12 h day lengths. In addition, sprays of the cytokinin 6-benzyladenine (6-BA) were unable to reduce the vernalization requirement of the winter genotype Urambie. The present study provides baseline data for plant growth regulator treatments that delay cereal development. These treatments might be extended in field studies to align flowering of early sown crops to the optimal flowering period.

## Introduction

Flowering time in cereal crops is one of the most important determinants of final grain yield. For crops to maximize seed size and number (potential yield), cereals must first establish, develop biomass and then flower at a time that coincides with optimal seasonal conditions (Fischer, 1985; Trethowan, 2014; Sadras and Dreccer, 2015). In the southern Australian cropping zone, flowering generally occurs in spring, when temperatures are starting to increase but overnight temperatures can still drop to <0°C with less cloudy days and rainfall. This means that flowering time potentially coincides with a number of environmental stresses that reduce grain yield, including frost (Marcellos and Single, 1984; Fuller et al., 2007), heat (Saini and Aspinall, 1982; Tashiro and Wardlaw, 1990) and drought (Saini et al., 1984; Del Moral et al., 2003). There is, however, a period when the risk of heat and drought as well as the risk of frost are at their lowest, which is commonly referred to as the optimal flowering period (OFP) when grain yield is on average maximized (Flohr et al., 2017). The dates and length of the OFP vary in different locations within the cropping zone due to climatic conditions; these have been well defined in wheat (Flohr et al., 2017), but to a lesser extent in barley (Liu et al., 2020).

Flowering time in cereals is under strong Genotype × Environment × Management (GxExM) control, where major crop development genes have a large influence. Briefly, *VERNALIZATION 2* (*VRN2*) represses flowering until a build-up of cold temperatures (vernalization) slightly increases *VERNALIZATION 1* (*VRN1*) expression; under long days *VERNALIZATION 3* (*VRN3*) further promotes *VRN1* leading to the acceleration of flowering (Distelfeld et al., 2009; Oliver et al., 2009). The photoperiod locus *PHOTOPERIOD D1* (*Pdp-D1*) upregulates *VRN3* [the barley homolog of *FLOWERING LOCUS T1* (*FT1*) in Arabidopsis] under long days, or continuously for photoperiod insensitive varieties depending on the allelic combination (Eagles et al., 2009). Once *VRN1* and *FT1* have been upregulated, earliness *per se* (*EpS*) genes determine the transition to flowering (Appendino et al., 2003). These *EpS* genes are regulated by complex interactions with temperature from different allelic combinations (Slafer and Rawson, 1995), which regulate the final time to flowering.

The current management strategy to ensure that flowering occurs within the OFP aligns the sowing date with a variety with the correct phenology. However, late opening rains delay germination, while seasonal temperatures and climate variability determine when flowering actually occurs (Sadras and Monzon, 2006; Pook et al., 2009). This reduces the control growers have over flowering and often means it occurs outside the OFP, lowering grain yield potential. Another constraint is that farming operations are generally getting larger, which means growers have to sow earlier, thus increasing the overall sowing date range. This makes it challenging for growers to match flowering to the OFP without having a number of varieties with different development speeds. In mixed farming systems, growers can use grazing to slow development and delay flowering to a certain extent (Virgona et al., 2006). Currently there is no option for accelerating development in season to compensate for any delay in sowing, which may push flowering past the OFP. This highlights the need for other management options to manipulate development and flowering time in barley and other cereals.

One possibility to manipulate development, which is already used in the horticultural industry, is the use of exogenously applied plant hormones or plant growth regulators (PGRs). Plant hormone research has been conducted for over 80 years with documented impacts on growth and development in a range of agriculturally relevant plants (for recent cereal-related reviews see Van De Velde et al., 2017; Marzec and Alqudah, 2018; Izawa, 2021; Kosakivska et al., 2021). In cereals, PGRs are a viable management option and are extensively used in high rainfall areas to prevent lodging (Peake et al., 2020). A number of studies have suggested that exogenous application of plant hormones can alter development and the time to flowering (Razumov, 1960; Barabas and Csepely, 1978; Al-Jamali et al., 2002; Pearce et al., 2013), but whether this might be co-opted to adjust the OFP remains unclear.

Gibberellic acid (GA) is a candidate hormone that can potentially alter plant development. Pioneering studies found that flowering was not promoted solely by GA (James and Lund, 1960; Razumov, 1960). However, when GA was applied via seed soaking with a vernalization treatment, the onset of flowering could be accelerated by up to 10 days in wheat possessing a winter growth habit. This indicates that after the vernalization requirement is fulfilled and the switch from a vegetative to reproductive state has occurred, GA can accelerate the time to flowering. Consistent with this, weekly exogenous GA applications to the roots of barley promoted flowering (Boden et al., 2014) once the plants had progressed toward reproductive development. Long days also cause an upregulation of GA biosynthesis genes to promote floral development in winter genotypes (Winfield et al., 2009; Pearce et al., 2013). This switch to long days has been correlated with increased endogenous GA levels (Vince-Prue, 1975). In the absence of endogenous GA under short days, even in photoperiod insensitive varieties (Pearce et al., 2013) there is a possibility that exogenous GA could hasten floral initiation. This has been shown in spring barley where the onset of flowering was accelerated by 10 days under short day lengths compared to the untreated controls (Cottrell et al., 1982). However, this effect could not be replicated under long days where GA was applied via a small solution through a scratch in the most advanced seedling leaf. Using the same scratching method of application, GA also promoted floral initiation in isogenic dwarf and tall Mexican spring wheat varieties under short days (Evans et al., 1995). Evans et al. (1995) also described a positive effect on flowering when increasing the concentration of different bioactive GAs, with GA3 being the most effective.

Endogenous GA activity can be regulated by exogenous application of compounds that inhibit GA biosynthesis. PGRs such as paclobutrazol and trinexapac-ethyl regulate different stages of the GA biosynthesis pathway and exogenous application can reduce the rate of active GA biosynthesis within the plant (Rademacher, 2000). Early studies with GA inhibitors in wheat showed an increase in the time to ear emergence of up to 8 days with a spray application at the six leaf stage, with the highest concentration having the largest effect (Humphries et al., 1965). Other studies reported a delay in ear emergence with a single spray at tillering (Lowe and Carter, 1972). The most extensive study performed to date was on a number of Mexican wheat varieties that involved different timings of spray applications between early stem elongation and flag leaf emergence. The results showed a delay in flowering by up to 5 days (Grijalva-Contreras et al., 2012). As a GA-inhibitor, trinexapac-ethyl is commonly used in high rainfall cereal growing regions beyond the onset of stem elongation (GS30) to mitigate lodging. However, the analysis of the effect on flowering time in current elite varieties with different genetic flowering controls is not well documented.

Cytokinins (CK) are another group of hormones that can influence development. A range of synthetic and naturally occurring CKs have been used across different growth and development studies. For example, kinetin, a naturally occurring CK, was able to reduce the time to flowering of winter wheat (Barabas and Csepely, 1978). A 20 day vernalization treatment with the addition of kinetin was able to replicate the same heading percentage achieved by 40 days of vernalization, effectively halving the required vernalization time (Barabas and Csepely, 1978). The reduction in the vernalization requirement was also replicated with the use of the synthetic CK 6-benzyladenine (6-BA) but to a slightly lesser extent (Csepely and Barabas, 1979). Both of these studies used seed soaking with exogenous kinetin and 6-BA mixed in solution to germinate seeds during a vernalization period of varying length. In addition, Pogna (1979) demonstrated that long day vernalization with an application of exogenous kinetin accelerated the overall time to flowering compared to short day vernalization. This interaction of CKs with the vernalization pathway of cereal plants has been linked to changes in endogenous CK levels within vernalizing winter cereals (Reda, 1976). In field studies, exogenous application of CK to winter wheat at double ridge has been suggested to reduce the vernalization period; however, no quantification of the actual time to flowering was shown (Shourbalal et al., 2019). Moreover, the impact of CKs on reducing the vernal requirement of winter cereals has yet to be confirmed or replicated with exogenous spray applications on plants in the early vegetative/vernalizing period of development.

Ethylene can also have secondary effects on development through interaction with the GA signaling pathway. The application of ethylene promotes DELLA protein activity (Achard et al., 2003), which effectively inhibits GA signaling. This has led to similar effects to other GA inhibitors where flowering was delayed by spray applications (Al-Jamali et al., 2002). Other hormones such as abscisic acid and jasmonic acid potentially promote flowering (Hall and McWha, 1981) and inhibit flowering (Diallo et al., 2014), respectively. These hormones require further investigation into the extent that they can influence cereal crop development, especially in different genetic backgrounds.

In summary, the use of PGRs to manipulate development has been adopted in a number of perennial horticultural crops (Nickell, 1994), but to a lesser extent in broadacre agriculture. Therein lies an opportunity to develop a new system for growers to better match their flowering time to the OFP to maximize grain yields. However, for a PGR application to be used in a broadacre context, the potential difference in flowering time needs to be quantified and optimized in relevant varieties. Therefore, the aim of this study was to manipulate crop development by exogenous spray applications of PGRs to current elite Australian barley varieties representing distinct genetic backgrounds. Knowledge from such experiments could lead to the identification of the optimal timing and concentration of the applied products without severely altering plant growth, and inform similar studies using international germplasm that may be susceptible to changing climatic conditions. A secondary aim was to investigate the GxExM interactions of exogenous PGR applications on flowering time and phases of plant development. Overall, the outcomes of this research form the foundations of extension materials to facilitate enhanced adoption of better management practices in the field, and for further genetic research into the hormone-sensitivity of flowering in different cereal species and varieties.

## Materials and Methods

### Experiment 1 – Screening of Different Plant Hormones for a Developmental Response

#### Plant Material, Flowering Genetics, and Growing Conditions

Experiment 1 tested 10 plant hormone products and PGRs (Table 1) for plant development responses. Four genetically distinct elite spring barley varieties were selected including RGT Planet, Compass, Schooner and Spartacus CL. Schooner is a historical Australian variety while the other three varieties have current market dominance in southern Australia and a range of different spring flowering times (GRDC, 2020). The selected varieties represent 70% of the Australian barley crop, a range of diverse genetics (Supplementary Table 1), and almost 30% of the world’s export-quality malt barley. In addition, RGT Planet represents a genotype of significant global interest based on its cultivation on multiple continents. Information regarding allelic differences in the major flowering genes is provided in Supplementary Table 1.

**TABLE 1:** Summary of the different plant hormones and PGR products used in Experiment 1 with their respective active ingredient and concentration.

Active ingredient	Hormone/Mode of action	Trade name	Spray concentration	Registered in cereals?
*250 g L^–1^ Paclobutrazol*	Gibberellic acid inhibitor	KULT	400 mL/100 L	No
*400 g kg^–1^ Gibberellic acid*	Gibberellic acid	ProGibb SG	80 g/100 L	No
*250 g L^–1^ Trinexapac-ethyl*	Gibberellic acid inhibitor	Moddus Evo	400 mL/100L	Yes (post GS30)
*19 g L^–1^ Gibberellins A4* + *A7 and 19 g L^–1^ 6-Benzyladenine*	Gibberellic acid and Cytokinin	Upcell	2 L/100 L	No
*20 g L^–1^ 6-Benzyladenine*	Cytokinin	Abscission	5 L/100 L	No
*0.075 g L^–1^ NAA and 0.075 g L^–1^ Indole acetic acid*	Auxins	Auxinone	5 L/100 L	No
*20 g L^–1^ NAA*	Auxin	N.A.A Stop Drop	500 mL/100 L	No
*100 g kg^–1^ Prohexadione-calcium*	Gibberellic acid inhibitor	Prohex	70 g/100 L	No
*8.84 g L^–1^ Indole-3-butyric acid*	Auxin	Radiate	300 mL/100 L	Yes
*99% pure methyl jasmonate*	Jasmonic acid	Methyl Jasmonate	0.4 mL/100 L	No

*Whether they are on label and registered for use in cereals is also listed.*

The plants were grown in a glasshouse during August and September in Adelaide, South Australia, under photoperiods of 10–12 h. The glasshouse followed a temperature regime of 23/20°C day/night cycles. Plants were grown in olive pots (17 cm by 7.5 cm by 7.5 cm) where three seeds were sown into coco peat at 2 cm soil depth. The coco peat mixture contained a 1:1 ratio of coir fiber to quarried drainage sand and controlled release fertilizer. From the three seeds, one plant was selected from each pot to grow to maturity across each of the replicates for that variety. The pots were watered with rainwater to field capacity. Plants were supplied with a standard rate of a commercially complete slow release fertilizer (25:5:8.8 NPK) applied as a liquid to the soil at the end of tillering (GS30).

#### Treatments and Design

Each variety was screened with a different hormone or PGR (Table 1). Each product was tested at two time points early in development; the first spray was applied at three leaf (GS13) and the alternative spray was applied at the identification of the first node (GS31) according to Zadoks et al. (1974). The hypothesis was that either an early application during leaf development or later application at the start of stem elongation could potentially allow for a response in development during vegetative or reproductive development. High rates of PGR application (Table 1) were determined from label rates and literature, and used to facilitate a developmental response for screening. A control sprayed with water was included as a standard for comparison. Experiment 1 was organized into a randomized complete block design, with four replicates where each replicate was blocked onto different benches in the glasshouse.

The chemical spray treatments were applied using a spray pump bottle to plants that were growing optimally. A solution of each hormone product was prepared in separate bottles using reverse osmosis water. The methyl-jasmonate solution also contained 4% ethanol to ensure even absorption on application. Each plant was sprayed with the solution thoroughly until the solution was running off the leaves, to replicate a broad acre spray application. The number of spray pumps for each plant was recorded as well as a calibration of the amount sprayed per pump to determine the amount of product applied; this equated to a total spray volume for each plant of approximately 5 mL.

#### Measurements

Flowering date was the only measurement. This is sometimes a confusing measure, since heading date, awn tipping and anther extrusion are sometimes used interchangeably but incorrectly (Alqudah et al., 2014; Alqudah and Schnurbusch, 2017). In Experiments 1 and 2, the flowering date was determined by scoring when the main stem had reached awn tipping (also referred to as awn peep) for easy assessment in the initial screening stage. In Experiment 3 (see below) both awn tipping and anther extrusion were scored.

### Experiment 2 – Development of Concentration Response Curves for Selected Plant Growth Regulator Products

Two varieties were chosen for this experiment. Spartacus CL was used as the ‘model’ photoperiod sensitive spring variety for the GA and trinexapac-ethyl response curves, while Urambie was used as the model photoperiod insensitive winter type variety to determine the vernalization response to 6-benzyladenine. Six concentrations (Table 2) were used to produce a concentration response curve with the aim of determining the optimal concentration for the three ‘model’ hormone/PGR products. Based on results from Experiment 1 and background literature, curves were developed for GA (ProGibb), trinexapac-ethyl (Moddus Evo) and 6-benzyladenine (Abscission) with optimal rates determined by assessing the maximum change in developmental speed without completely compromising growth. Two timings of application were tested on Compass with one at GS13 and the other at GS33 for GA and trinexapac-ethyl. 6-benzyladenine was only applied once at GS13 on Urambie.

**TABLE 2:** The three ‘model’ hormone products with the six rates and molecular concentrations selected to generate a concentration response curve.

*Product*	*Rate 1*	*Rate 2*	*Rate 3*	*Rate 4*	*Rate 5*	*Rate 6*
*Gibberellic acid (mg/L)*	0	10	50	100	250	500
*Trinexapac-ethyl (mg/L)*	0	100	250	500	1000	5000
*6-Benzyladenine (mg/L)*	0	10	25	75	150	1500

Pots and growing media were the same as per Experiment 1. Plants were grown in a controlled growth room (CER) under a 16 h day length with 22°C days and 8°C nights, to allow for vernalization and temperatures that were generally representative of Southern Australian growing environments. Lighting was generated using 10 individual 400 watt high pressure sodium lights spread out above the benches at a height of 1 m. The temperature was recorded using a *Tinytag TPG-4017* data logger at plant height. Temperature accumulation was determined by averaging the temperature for the room for a 24 h period to form the degree days or thermal time accumulated from sowing to flowering. In addition to the standard flowering time measurements, height was recorded at physiological maturity.

Experiment 2 used a randomized complete block design. Four replicates were blocked into rows perpendicular to the wall in the CER. The trinexapac-ethyl, GA and 6-benzyladenine concentration experiments were all conducted separately on different benches in the CER.

### Experiment 3 – Developmental Responses of Different Barley Varieties to Gibberellic Acid, Trinexapac-Ethyl and 6-Benzyladenine Under Different Day Lengths and Vernalizing Treatments

The three varieties used for Experiment 3 were Spartacus CL (photoperiod sensitive spring-type), Urambie (winter-type) as previously described, along with Compass (photoperiod insensitive spring-type). The three varieties were chosen to represent contrasting flowering controls to investigate the effect of hormones on the different flowering pathways. Seeds in this experiment were sown into soil either with or without (see below) a prior vernalization treatment.

Plants were grown in two CERs using the same pot media and light intensity as Experiment 2. One room was set to long day conditions of 16 h at 22°C under light, and 8 h at 8°C with no light. A second room was programmed to short day conditions, with an 8 h day length and the same temperature regime. Thermal time was calculated as per Experiment 2 to form a standard measure of development. The 8°C night cycles reflect mild winter conditions and were used to gradually fulfill the vernalization requirement of Urambie. This gradual loss of flowering repression allowed us to assess the impact of hormone treatments (e.g., CK) to hasten the transition to reproductive development/flowering during the growing period.

The concentrations for the three hormone products were selected based on the concentration response curves. These were chosen to maximize the change in flowering time while not completely comprising growth. The timing of the spray was determined by the results from Experiment 2, with the criteria being to spray at the earlier plant development stage if the later application did not produce a more significant change in the time to flowering. GA and 6-BA were sprayed at 100 mg/L, while the trinexapac-ethyl rate was 1000 mg/L with the spray timing being a single spray at the three leaf stage.

Experiment 3 was a split plot design where each whole plot was variety, subplot vernalization and plot level being a PGR treatment. Two replicates were situated on each side of the CER to make up a total of four replicates, randomized in rows away from the walls on each side. The day length factor was confounded by only having one CER for long and short days.

#### Vernalization Treatment

A 6 week vernalizing treatment was carried out on germinated seeds in a petri dish with the aim of saturating the vernalization requirement prior to sowing. This enabled Urambie to rapidly transition to flowering with its vernalization requirement already satisfied, and allowed us to investigate the effect of hormone (e.g., GA) treatments on development when the vernalization requirement was already fulfilled.

The 6 weeks vernalizing treatment was performed by germinating seed in a fridge set at 4°C. Firstly, 50 seeds for each variety were placed on 1 piece of filter paper and sealed in a petri dish with parafilm. Water was added to approximately 50% water by weight of the seeds. The sealed petri dishes were left at room temperature for 24 h to allow them to imbibe. They were then placed in the fridge for the 6 weeks period before being sown straight into pots in the growth rooms. Two petri dishes were prepared for each variety of the same seed source to allow for selection of the most uniform seeds to sow.

#### Measurements

For Experiment 3, growth stages (GS) were assessed on a daily basis using Zadoks growth scale (Zadoks et al., 1974). Three leaf stage (GS13) was recorded when the third leaf reached the length of the second leaf. The switch from vegetative to reproductive growth was determined when the first visible node was present on the main stem of the plant (GS31), which could be assessed by visual and manual examination. Awn tipping/awn peep (GS49) was determined by the presence of awns protruding out of the flag leaf ligule. We also assessed when the first anthers on the main stem had turned yellow with approximately 50% anther extrusion (GS60). Plant height was measured from the base of the stem to the tip of the emerged spike (excluding awns). A progressive height measure was taken at GS13, GS31, and GS49.

### Design and Data Analysis

The change in the time to flowering across different genotypes and hormone treatments was analyzed using an ANOVA in statistical package GenStat for Windows (2018) 19th ed. (VSN International Ltd., Hemel Hempstead, United Kingdom), which was used for the analysis of all experiments. Differences among treatment means were examined by a 5% least significant difference (LSD), with the treatment structure being variety × PGR for Experiment 1. Experiment 2 had each product analyzed separately in GENSTAT, where the treatment structure was concentration × timing. A Bonferroni test was used for multiple comparisons of concentrations for each product. Finally, Experiment 3 had long and short day rooms analyzed separately. The treatment structure was variety × vernalization × PGR where contrasts were made for comparisons of different PGRs to the control.

## Results

### Effect of Different Plant Growth Regulators Products on Barley Flowering Time

There was a significant variety × PGR interaction (*p* = 0.04; Table 3). GA4 + GA7 and 6-benzyladenine, prohexadione-calcium and indole-3-butyric acid treated materials were significantly faster in their time to flowering only in Compass; although compared to the control it was only by 2, 2 and 3 days respectively. GA3 also marginally reduced the time to flowering by 2 days for Schooner and Compass. Methyl Jasmonate, trinexapac-ethyl and paclobutrazol were the only products to slow the time to flowering in almost every variety, except paclobutrazol in Schooner. Methyl Jasmonate delayed flowering the most across varieties (on average by 5 days), however, widespread chlorosis/defoliation of leaves was noted in days following the spray application with new shoots developing after leaf death.

**TABLE 3:** The relative number of days to flowering for different barley varieties in the glasshouse when sprayed at GS13 and GS31 with different plant growth regulators (PGRs).

*PGRs*	*Compass*	*Planet*	*Schooner*	*Spartacus CL*
*Control*	53.3	52.0	49.5	48.8
*Methyl jasmonate*	58.8*	58.5*	54.0*	56.5*
*Trinexapac-ethyl*	57.5*	55.7*	53.3*	54.3*
*Paclobutrazol*	57.0*	53.8*	50.8	51.0*
*6-Benzyladenine*	52.8	52.3	49.5	49.3
*NAA and Indole acetic acid*	52.0	52.3	49.0	48.8
*NAA*	51.8	52.0	49.0	48.5
*GA4 + GA7 and 6-Benzyladenine*	51.5*	52.0	49.8	48.3
*GA3*	51.3*	51.8	47.3*	47.5
*Prohexadione-calcium*	51.0*	51.0	48.3	48.2
*Indole-3-butyric acid*	50.5*	51.8	50.3	48.5
*Variety × PGR (LSD 5%)*	1.62			

*Flowering time data was analyzed by ANOVA in statistical package GENSTAT.*

*Indicates that the PGR treatment on that variety was significantly (LSD 5%) different to the control.

### Development of Concentration Response Curves for Gibberellic Acid, 6-Benzyladenine, and Trinexapac-Ethyl

**FIGURE 1:**
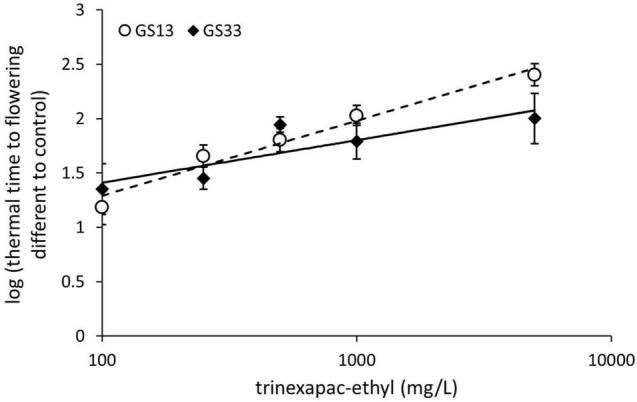
The concentration response curve for trinexapac-ethyl with one spray application at three leaf stage (GS13) or GS33 with five different concentrations in Spartacus CL. Each fitted with a logarithmic trend line where error bars are representative of one standard error of the mean of four replicates.

The concentration response curves for trinexapac-ethyl applied at the chosen growth stages of three leaf (GS13) and stem elongation (GS33) are shown for Spartacus CL in Figure 1. The effect of trinexapac-ethyl concentration on time to flowering was significant (*p* < 0.001; Table 4). Generally, as the concentration of trinexapac-ethyl increased, the time to flowering also increased. However, the early application (GS13) produced a similar delay in flowering compared to the late stem elongation spray (GS33) at the different concentrations. This is reflected in the statistical analysis that showed there was no significant effect of timing (*p* = 0.12) or the interaction between timing × concentration (*p* = 0.11).

**TABLE 4:** The significance of concentration, timing and their interaction on the thermal time to flowering and final height in Spartacus CL after spray applications of trinexapac-ethyl, 6-benzyladenine, and gibberellic acid.

	*Thermal time to flower*			*Final height*	
	*Trinexapac-ethyl*	*6-Benzyladenine*	*Gibberellic acid*	*Trinexapac-ethyl*	*Gibberellic acid*
*Concentration*	<0.001	0.111	0.13	<0.001	<0.001
*Timing*	0.116	–	0.178	0.001	<0.001
*Concentration.Timing*	0.107	–	0.285	<0.001	0.027

*Flowering time and height was analyzed by ANOVA in statistical package GENSTAT at the 5% significance level from four replicates.*

The application of GA3 to Spartacus CL and 6-BA to Urambie had no significant impact on the time to flowering at any concentration (GA3, *p* = 0.13; 6-BA, *p* = 0.11; Supplementary Figures 1, 2).

**FIGURE 2:**
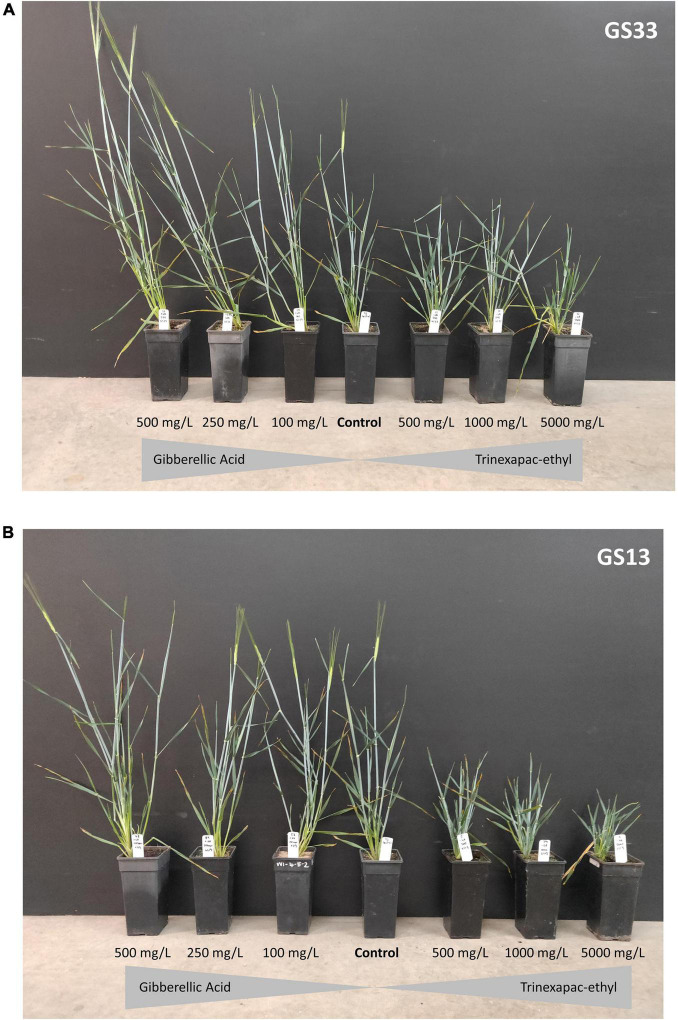
A visual representation showing the effect of concentration and timing on the height and development speed of Spartacus CL with a spray application of gibberellic acid and trinexapac-ethyl at (A) GS33 and (B) GS13. One representative sample from four replicates at the three concentrations of each product are shown.

**FIGURE 3:**
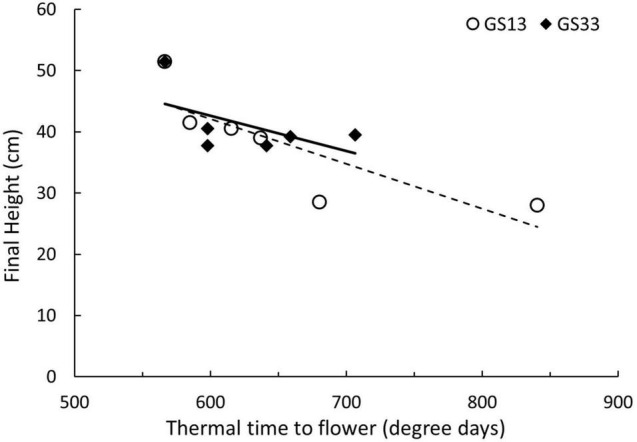
Changes in height and flowering time in Spartacus CL from a spray application of trinexapac-ethyl at GS13 and GS33. Each point represents a concentration of trinexapac-ethyl with its respective thermal time to flower and final height.

Final height was significantly different across concentration and timing for both trinexapac-ethyl and GA3 (Table 4). Figure 2 and Table 5 show the effect of increasing concentration of each GA-related product. Notably, later application (GS33) of GA3 significantly increased height by approximately 15 cm in comparison to the control (Figure 2), but earlier applications (GS13) had no significant impact. For trinexapac-ethyl treatments, changes in height coincided with a delay in development and thermal time to flower, and showed a negative correlation (Figure 3). The early application only showed a moderately strong negative linear relationship with final height. For every 15 degree days delay (approximately equivalent to 1 day in the field during early spring) in flowering time, height was reduced by 1 cm. 6-BA had no significant effect on the height of Urambie.

**TABLE 5:** The final height and thermal time to flowering produced from two different timing applications of **(a)** trinexapac-ethyl and **(b)** gibberellic acid.

Trinexapac-ethyl (mg/L)	GS13 (cm)	GS13 (degree days)	GS33 (cm)	GS33 (degree days)	Gibberellic acid (mg/L)	GS13 (cm)	GS13 (degree days)	GS33 (cm)	GS33 (degree days)
*0*	51^a^	567^a^	51^a^	567^a^	*0*	51^a^	567^a^	51^a^	567^a^
*100*	42^b^	585^ab^	41^b^	598^ab^	*10*	48^a^	546^a^	53^a^	568^a^
*250*	41^b^	615^ab^	38^b^	598^ab^	*50*	52^a^	559^a^	56^a^	572^a^
*500*	39^b^	637^ab^	39^b^	641^bc^	*100*	52^a^	546^a^	56^a^	550^a^
*1000*	29^c^	680^b^	38^b^	659^bc^	*250*	52^a^	563^a^	67^b^	559^a^
*5000*	28^c^	841^c^	40^b^	706^c^	*500*	54^a^	555^a^	68^b^	550^a^
	*p* < 0.001	*p* < 0.001	*p* < 0.001	*p* < 0.001		*p* = 0.21	*p* = 0.16	*p* < 0.001	*p* = 0.34
									

*Height and time to flowering were analyzed by ANOVA in statistical package GENSTAT at the 5% significance level from four replicates, with multiple comparisons made between concentrations at each growth stage through a Bonferroni test. Super script letters represent significance with overall significance (p-value) for each timing and product represented at the bottom.*

The selected concentration for trinexapac-ethyl for Experiment 3 was determined by assessing the maximal delay in development that did not completely jeopardize growth or biomass production. This chosen concentration (1000 mg/L of trinexapac-ethyl) also happened to be the label rate for commercial application in the field to prevent lodging. With no significant difference in flowering time for GA3, the concentration of 100 mg/L was determined by the highest label rate of the product for commercial use, and previous studies (Boden et al., 2014). The same selection criteria were used for the concentration of 6-BA (100 mg/L) which was again a combination of high label rate and previous studies (Barabas and Csepely, 1978).

### Developmental Responses of Different Barley Varieties to Gibberellic Acid, Trinexapac-Ethyl and 6-Benzyladenine Under Different Day Lengths and Vernalizing Treatments

In Experiment 3, plant development and flowering time responses to selected PGRs were assessed at different daylengths. As expected, significant (*p* < 0.001) varietal differences were identified in time to GS31 and time to flowering (Table 6).

**TABLE 6:** Statistical analysis of interactions between day length, variety, vernalization, and PGR treatment in Experiment 3.

	Short days		Long days	
	*Thermal time to GS31*	*Thermal time to flower*	*Thermal time to GS31*	*Thermal time to flower*
Variety	<0.001	<0.001	<0.001	<0.001
Vernalization	0.27	0.09	<0.001	<0.001
PGR	<0.001	<0.001	0.41	0.10
Control vs. 6-BA	0.6	0.77	0.67	0.5
Control vs. GA	0.18	0.77	0.67	0.42
Control vs. TRE	<0.001	<0.001	0.23	0.09
Variety.PGR	<0.001	0.06	0.02	0.28
Control vs. 6-BA	0.12	0.67	0.96	0.85
Control vs. GA	0.94	0.68	0.72	0.54
Control vs. TRE	<0.001	0.06	0.02	0.29
Vernalization.PGR	0.13	0.1	0.09	0.49
Variety. Vernalization.PGR	0.58	0.57	0.25	0.25

*Short day and long day analysis were carried out separately with thermal time being the main developmental measure for comparison. Thermal time data was analyzed by ANOVA in statistical package GENSTAT at the 5% significance level from four replicates. Contrasts to the control are displayed, demonstrating the difference of the PGR compared to the control replicate.*

Under long days, there was a significant effect of vernalization, but no PGR × vernalization effect. In addition, a PGR × variety effect was significant for thermal time to GS31, but not for time to flowering. The PGR effect alone was not significant, despite trinexapac-ethyl delaying the time to GS31 by up to 300 degree days in Compass. This analysis was partly compromised by variability introduced from the Urambie treatments. The non-vernalized Urambie replicates did not acquire sufficient vernal time under long days to make the switch to stem elongation. This also affected the vernalized Urambie plants, causing some plants to flower while others were delayed. To account for this, another analysis is supplied in the Supplementary Table 2 excluding Urambie, which demonstrates that the delay of trinexapac-ethyl of up to 300 days is significant.

**FIGURE 4:**
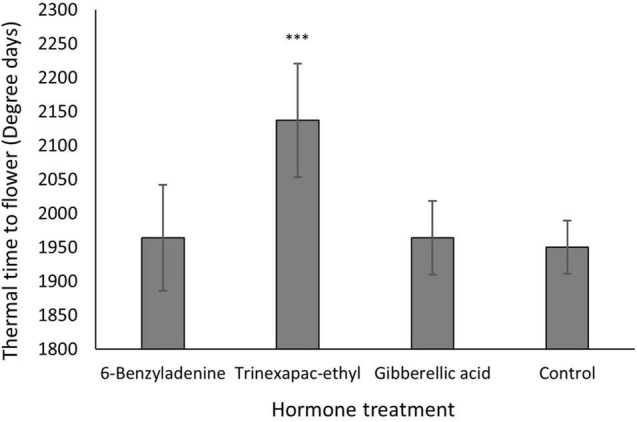
Thermal time to flowering across different hormone treatments from one spray application at GS13 for all varieties under 8 h day lengths. Thermal time data was analyzed by ANOVA in statistical package GENSTAT at the 5% significance level. ^***^P < 0.001 (different compared to control), where error bars represent one standard error of the mean difference.

Under short days, no significant effect was identified for vernalization or vernalization × PGR. In contrast, the PGR × variety interaction was significant for thermal time to GS31, but not for time to flowering. Consistent with this, Figure 4 shows the PGR effect on thermal time to flower is essentially variety-independent. The clear delay induced by trinexapac-ethyl was significant (*p* < 0.001) when compared to the control and is roughly equivalent to 200 degree days or a 10% increase in the time to flowering.

**FIGURE 5:**
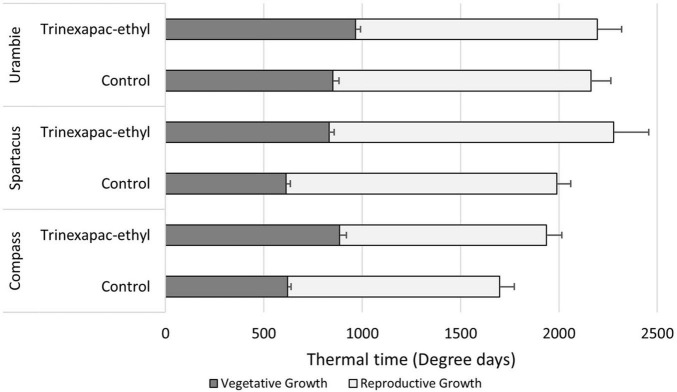
The amount of thermal time accumulated during the vegetative (sowing to GS31) and reproductive (GS31* to GS60) growth phases under short days. The variety × PGR interaction is shown with different varieties and their respective differences that trinexapac-ethyl had on development in comparison to the control. Error bars represent one standard error of the mean difference for the time to GS31 and GS60 respectively. *GS31 was used as a proxy for the start of rapid inflorescence development, but not necessarily the very beginning of reproductive development.

The delay in flowering time with an early (GS13) application of trinexepac-ethyl under short day conditions comes prior to GS31 (Figure 5). This difference is maintained all the way until anther extrusion (GS60). The difference compared to control plants did not increase post-GS31, but the standard error of the means did increase at GS60. Trinexapac-ethyl changed thermal time to GS31 but the efficacy was somewhat dependent on variety. Figure 5 shows this, where the relative duration of the vegetative and reproductive growth phases of each variety are displayed under short day lengths. All varieties had a significant (*p* < 0.001) delay in timing of GS31 with trinexapac-ethyl applications; however, the magnitude of the response determined whether it was still significant at flowering time. Urambie was delayed, but the difference at flowering was not significant with a similar overall thermal time to flowering. Compass still maintained the trend of the trinexapac-ethyl treatment, delaying the time to flowering but the length of the reproductive growth phase was similar in thermal time compared to the control. This pattern was similar for Spartacus CL, where the vegetative phase with the PGR was significantly longer than the control but the reproductive phase was similar. Spartacus CL, being a photoperiod sensitive variety, struggled to transition to reproductive growth under short day lengths which delayed flowering time. Trinexapac-ethyl made this effect even more significant and variable with the standard error of the mean quite large when compared to the other varieties.

## Discussion

This study demonstrates it is possible to delay barley development and flowering time using exogenous applications of PGRs. While we also observed several examples of accelerated crop development after PGR treatment, results were variable, and the effect was relatively minor. The changes in development showed significant GxE interactions in elite spring barley germplasm, suggesting there may be potential for targeted breeding to develop varieties more responsive to exogenous PGR application. Independent of this, the delay to flowering induced by PGR treatment that exceeded approximately 10 days is a key finding; with further agronomic testing this could quickly be adapted to field practices.

### Gibberellin Plays a Key Role in Flowering

Previous genetic studies have highlighted the importance of gibberellins in the regulation of flowering (Boden et al., 2014). Here, the influence of gibberellins on flowering was demonstrated through the GA-inhibiting compounds paclobutrazol and trinexapac-ethyl. Each of these PGRs delayed flowering time across spring barley varieties (Table 3), with observed differences in height and delayed flowering similar to those reported previously (Humphries et al., 1965; Grijalva-Contreras et al., 2012). However, when GA3 was applied, there were only small, inconsistent differences in flowering time that were variety dependent. Compass and Schooner were the only varieties that were significantly faster than the control. In previous studies, exogenous GA3 applications have been shown to promote apical growth (Cottrell et al., 1982; Boden et al., 2014) and floral development (Mutasa-Göttgens and Hedden, 2009; Pearce et al., 2013), most likely through GA-sensitive floral identity genes. The varied response identified here may be indicative of the significant variety × PGR interaction (*p* = 0.041). Different genotypes likely have different levels of endogenous GA production or metabolism, resulting in varied responses to exogenous GA applications and floral promotion. This suggests there could be genetic potential to breed varieties more receptive to exogenous hormone treatments to have greater control of flowering.

To further investigate this GxE interaction with GA, a concentration curve was developed and then selected genotypes were examined under short and long day conditions. The use of GA3 as a spray application did not significantly promote flowering at any concentration or timing in Spartacus CL under 16 h days (Experiment 2). The same result was found for different varieties and at short day lengths (8 h; Experiment 3). This is contrary to results from Cottrell et al. (1982) who noted that Clipper barley treated with GA3 reached spike development stage 9 (Zadoks GS31-33) 10 days earlier than the untreated control. In our study, this increase in the rate of development was not observed in thermal time to GS31 or overall time to flowering. Pearce et al. (2013) also demonstrated a more advanced growth stage with spring wheat grown under short days with a GA application, as did Evans et al. (1995). However, eight separate applications of GA were applied over a 2 weeks period to achieve the desired promotion, which would not be sustainable if it was to be scaled up for use in broadacre operations.

Although there was no change in the speed of development after GA application, there were significant impacts on other traits such as plant height, suggesting exogenous GA3 is being absorbed by the plant. GA is known for its role in increasing stem elongation, resulting in significantly taller plants (Green, 1985). In Experiment 2, the later GS33 application had a greater effect on height than the GS13 application, as did higher rates of GA. This response is important, since changes in development through PGR application might lead to unintended effects that outweigh potential positive outcomes, i.e., plant height vs. modified flowering date. For example, with GA promoting stem elongation, taller plants may be more susceptible to lodging or head-loss in the field, resulting in lower grain yields. Other pleotropic effects that potentially influence the plant agronomically will also need to be analyzed before GA could be applied widely in the field.

**FIGURE 6:**
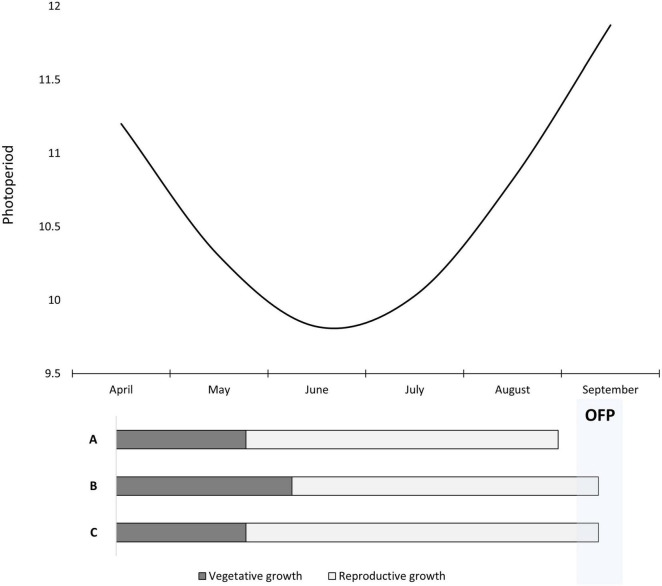
A schematic depicting day length for Adelaide, South Australia from April to September. Shown below is a representation of Spartacus CL sown too early in April with three different management scenarios. Scenario A is the control which experiences 11-h days in April, accelerating the transition to reproductive growth and causing flowering to occur before the OFP. Scenario B demonstrates early sowing but with an application of trinexapac-ethyl at GS13 which extends the vegetative growth phase and resulting in flowering occurring within the OFP. Scenario C has a late trinexapac-ethyl application at GS31 which extends the reproductive growth phase but results in flowering being delayed into the OFP as well.

Day length may play a significant role in the responsiveness to exogenous GA, but only under intermediate conditions. Compass and Schooner had significantly shorter times to flowering compared to the control in the glasshouse after exogenous GA application (Experiment 1). Performed in Adelaide, South Australia during August and September, the day length ranged from 10 to 12 h (Figure 6). Recent work in barley showed that *FT1* expression initiates under these day lengths as the transition to long days occurs (Gauley and Boden, 2020). *FT1* is a key driver of flowering initiation in cereals that activates important floral identify genes (Dixon et al., 2018). GA biosynthesis genes are day-length dependent (MacMillan et al., 2005; Boden et al., 2014), and GA regulates a range of flowering time genes in barley independent of *FT1* (Boden et al., 2014). Hence, during the day length transition from short to long days, there is potential for exogenous GA application to up-regulate flowering time genes to further accelerate floral development in the presence of *FT1* expression. However, further molecular and physiological research needs to be undertaken at different day lengths to determine if this is an explanation and viable solution to significantly accelerate the transition to flowering.

### Gibberellin Inhibitors Significantly Delay Flowering Time

Application of the GA inhibitor trinexapac-ethyl significantly delayed flowering time (*p* < 0.001) in all tested spring barley types and vernalized winter growth types. Importantly, trinexapac-ethyl is commercially available and used in crop production globally to reduce lodging in cereals. The delay in the time to flowering from germination ranged up to 200 degree days compared to the control when applied at 1000 mg/L. In field conditions, this delay equates to over 10 days during winter/spring months in Mediterranean conditions in southern Australia. This range is in agreement with other studies that found a delay in flowering with similar GA-inhibiting compounds of 5 to 10 days (Humphries et al., 1965; Grijalva-Contreras et al., 2012). There was no significant difference in the delay to flowering when GS13 and GS33 were compared. However, as shown in Figure 2, the early application at higher rates produced a greater delay, with the concentration being significant (*p* < 0.001), consistent with Humphries et al. (1965). This early application is significantly earlier than current practice for the prevention of lodging where sprays are applied post-GS30, and demonstrates an opportunity for growers to spray earlier than currently recommended, with the aim to delay flowering.

Along with the changes in flowering time, GA-inhibitors induced significant impacts on growth. Plant height was reduced with increasing concentration and differences in timing of application (*p* < 0.001). The timing interaction on the reduction in height is displayed in Figure 3, with the earlier application producing significantly shorter plants compared to the control. It must be noted that the change in flowering time was correlated to the increasing reduction in height which is shown in Figure 4. This suggests that in the tested cultivars, development cannot be altered independently of growth. A reduction in height for high rainfall zones where lodging is a problem would be a good management strategy. However, this may be a problem in low rainfall zones where rainfall and biomass production limit potential grain yield. The later application has less of an impact on height which might be an option for these low rainfall environments, however later applications of trinexapac-ethyl significantly increase sterility in the field (Zerner et al., 2015). For trinexapac-ethyl to be suitable for frost-prone environments, early applications will need to be used. This provides an opportunity for chemical companies to investigate applications at earlier stages than previously considered.

The early application of trinexapac-ethyl at GS13 was able to significantly delay the transition from vegetative to reproductive growth under short (*p* < 0.001) and long days (*p* = 0.02). This delay is effectively changing the different phases of growth as evident in Figure 6. The initial delay caused by trinexapac-ethyl is already 200 degree days to GS31, but does not increase further post-GS31, possibly due to chemical break down. This finding is consistent with work of Ma and Smith (1991) who found that another GA inhibiting product, chlormequat, reduced apical dominance and development early with a GS13 spray. Delaying the transition from vegetative growth as well as reduced apical dominance allows for more tillering and potential grain number before the switch to reproductive growth occurs (Ma and Smith, 1991). In contrast, a later mid-stem elongation application would extend the reproductive phase of growth where normally there are high rates of spikelet and floret abortion (Kirby, 1988; Fischer, 2007; Alqudah and Schnurbusch, 2014). This extended phase could allow for better partitioning of assimilates from reduced apical dominance, potentially explaining the increase in grain number observed by Ma and Smith (1991). Although this is speculative, it creates interesting scope for further yield and crop physiology research on the benefit of changing the duration of these critical periods leading up to flowering (Alqudah and Schnurbusch, 2017).

A delay of 200 degree days would produce a significant change in flowering time under field conditions in southern Australia and other Mediterranean climates. This sort of delay could be in excess of 10 days depending on the season. Figure 6 displays how a gibberellin inhibitor could work in delaying an early sown crop to better match the OFP. The interesting consideration is not just about better matching the flowering time to the OFP, but the interaction of long days on photoperiod sensitive varieties. With early to mid-autumn sowing, varieties often experience 10–11 h photoperiod inductive days (Figure 6) that switch on *FT1* and GA biosynthesis genes in photoperiod sensitive varieties (Gauley and Boden, 2020). This often causes early sown photoperiod varieties such as Spartacus CL to start reproductive development before the short days of winter, resulting in earlier flowering which causes problems with frost and low biomass production. GA inhibitors could be used in autumn on these photoperiod sensitive varieties to help prevent this earlier switch to reproductive growth, and to delay flowering time. In addition to the increased tillering and potential grain number, early sowing of photoperiod sensitive varieties could become a more viable option.

### Cytokinins and Vernalization in Winter Cereals

Based on the literature we hypothesized it may be possible to shorten the vernalization requirement of winter cereals with exogenous applications of CKs. Studies by Barabas and Csepely (1978) demonstrated that cytokinin seed soaking could in fact reduce the vernalization requirement of winter wheat, reducing the time to flower. A study by Reda (1976) found a correlation of rising endogenous CK levels during the vernalization process of winter wheat grains. However, few genetic studies demonstrating the linkage of CKs with vernalization in cereals beyond seed soaking have been reported. Although Shourbalal et al. (2019) suggested a reduction in vernalization time in the field, this was not supported with development/flowering time data. This reduction could not be replicated as an exogenous spray application of 6-benzyladenine to seedlings at any concentration. Under long or short days with 8°C nights in the CER, 6-BA did not produce any significant shortening of the vernalization requirement of the winter barley type of Urambie. In Experiment 3, there was no difference of the time in the vegetative phase of growth (thermal time to GS31) under short or long days, as well as the total time to flowering.

There was a high amount of variability in the non-vernalized Urambie in its time to flowering under controlled conditions. This variability was higher in the treated replicates in comparison to the control, suggesting that the hormone is being taken up. However, the plant may not be consistently responding to the exogenous CKs. To resolve this, gene expression of CK-responsive genes could be assessed and/or different seed sources or varieties should be tested to confirm that the variability is not related to spray application, off-types in the seed source, or varietal differences. In the interim, little interaction between CKs and vernalization is evident and therefore until consistent results can be demonstrated it is unlikely to have any commercial application.

### Other Hormonal Products May Have an Influence

The auxin indole-3-butyric acid also had some significant impacts on floral development. The combination of Compass and indole-3-butyric acid significantly reduced the time to flower, which was the largest reduction across all hormone treatments and varieties. This was only significant in Compass and with no other auxin products. This indicates that the genetic background and environment may be having a large influence on the response to these auxins. The role of auxins during cereal grain development has been discussed (Shirley et al., 2019), but their role during flowering in cereals is not well understood, and interactions with GA could be important. An earlier study showed that application of indole-3-butyric acid can increase the amount of bioactive GA present in the barley stem (Wolbang et al., 2004), with increased GA levels potentially producing a promoting effect. Another auxin, indole-3-acetic acid, has been shown to regulate particular components of the GA biosynthesis pathway in pea (Ross et al., 2000). Despite these results, further work needs to examine this GxE interaction to determine if this is a possible pathway to promote flowering in cereals.

Methyl jasmonate produced a large and consistent delay on flowering across all varieties. However, the rate used was likely too high to be solely a hormone effect, as in its most rudimentary form it is an acidic compound that causes widespread chlorosis and defoliation. The delay was derived from the plant having to produce new leaves and tillers after completely losing its original leaf area. Defoliation from grazing is a practice used in mixed farming systems to delay flowering by approximately 1 day for every 4–5 days grazing (Virgona et al., 2006). Chemical defoliation could work in a similar way with concentration and timing being the main controls to determine the extent of the delay in development. A surface contact chemical could be used to desiccate the leaves without killing the plant, working on a similar principle to grazing. However, no studies have addressed this idea and the potential for it to be a practical management option.

Finally, prohexadione-calcium which is a GA inhibitor sped up the time to flowering. As an inhibitor of GA, it would be expected to delay flowering. However, other studies in sorghum have found no significant delay to flowering (Lee et al., 1998); this was the case for most barley cultivars tested here, apart from Compass which had flowering promoted. One important consideration is whether other growth effects might be induced by specific PGRs, in addition to the significant GxE interaction. Prohex had a much lower rate of application compared to the other inhibitors and affects GA biosynthesis in another part of the complex biochemical pathway.

### Future Directions Within a Genetics × Environment × Management Framework

When we consider the GxExM framework, this study has provided additional research avenues to pursue. Genetically, it has been shown that there is potential for genotype-dependent differences in the responsiveness to applications of PGR products. This presents options to screen more genetically diverse germplasm for specific sensitivities. Screening of diverse cereal germplasm may also identify relevant genes that respond well to exogenous hormone/PGR applications. Variation in some cereal dwarfing genes’ sensitivity to GA have been shown to reduce height without reducing coleoptile length or seedling vigor (Rebetzke et al., 1999). This variation in GA sensitive dwarfing genes could also affect their sensitivity to exogenous GA applications and their impact on floral development genes. It could also help break the link found here that a delay in flowering time was not independent of reduced height with trinexapac-ethyl applications. Genetic differences could be investigated by undertaking a diversity panel screen. This could also be applied to the CK and vernalization interaction, where more diverse winter cereals should be screened for a more consistent response. Additionally, investigating the response of different bioactive GAs could be of use to promote developmental responses and not necessarily growth.

The environmental aspect is important for the interaction with photoperiod and vernalization. The CER experiments were performed under extreme day lengths which are not relevant to the natural day lengths in southern Australia. Further work needs to be done on day lengths of 10–12 h where *FT1* is starting to be expressed, which is key for flowering (Gauley and Boden, 2020). This goes both ways for the application of GA and GA inhibitors to speed up or delay flowering respectively. The period during autumn is where establishment date is dependent on opening rains, where crops may be too early or late in respect to development and flowering within the OFP. Different temperatures also need to be analyzed with respect to the duration these hormones are active in the plant. Warmer temperatures increase growth rates of plants, but does this mean the hormone product breaks down earlier? Further developmental stage applications could also be analyzed at different temperatures for maximum efficacy. In addition, testing during colder vernalizing temperatures may be required especially when examining the interaction of CKs and shortening the vernalization period.

This study has demonstrated the potential for PGRs to be used as a management option to better control cereal crop development. The most significant finding appears to be offered through the use of gibberellin inhibitors, which caused a significant delay of 200 degree days to flowering with early and late applications. The data provided by this study shows what might be achieved in controlled environments; the next phase is to replicate this in the field while analyzing the effects on overall grain yield. This could include spraying prior to GS30 which would be a change to the way current PGR products are used on farm. However, further studies into the agronomic trade-offs of spraying early need to be conducted to ensure they do not outweigh the benefits of delaying development. This leads into the potential option of changing pre-anthesis development phases with a management practice. Phase changes are being selected genetically by plant breeders to maximize yields by extending critical periods of growth such as tillering or just prior to flowering. PGRs could be another option to maximize grain yields by targeting these phases.

## Data Availability Statement

The original contributions presented in the study are included in the article/Supplementary Material, further inquiries can be directed to the corresponding author/s.

## Author Contributions

BK developed and executed the experimentation, data acquisition, analysis, interpreted results, and wrote the manuscript. KP formed the research idea, developed the experimentation, interpreted the results, drafted and edited the manuscript. MT and JA developed the experimentation, interpreted the results, drafted and edited the manuscript. All the authors contributed to the article and approved the submitted version.

## Conflict of Interest

The authors declare that the research was conducted in the absence of any commercial or financial relationships that could be construed as a potential conflict of interest.

## Publisher’s Note

All claims expressed in this article are solely those of the authors and do not necessarily represent those of their affiliated organizations, or those of the publisher, the editors and the reviewers. Any product that may be evaluated in this article, or claim that may be made by its manufacturer, is not guaranteed or endorsed by the publisher.
