# Supplementary material for: Manipulation of Barley Development and Flowering Time by Exogenous Application of Plant Growth Regulators
Source: Front Plant Sci. 2022 Jan 3;12:694424. doi: 10.3389/fpls.2021.694424 (PMC8761979; doi:10.3389/fpls.2021.694424)
Supplement: Supplementary file 1 [file Data_Sheet_1.docx]

Supplementary Material

# Supplementary Figures and Tables

**Table 1 The allelic state of the key flowering development genes in barley for the five varieties used in Experiment 1, 2 and 3. Data was provided by the GRDC project ULA00011 (T.Rathjen, J.Hunt, *pers. comm.*)**

| **Cultivar** | **Vrn-H1** | **Vrn-H2** | **Ppd-H1** |
| --- | --- | --- | --- |
| Compass | Vrn1-4 | DEL | Insensitive |
| RGT Planet | Vrn1-2 | DEL | Insensitive |
| Schooner | Vrn1-1 | DEL | Sensitive |
| Spartacus CL | Vrn1-4 | DEL | Sensitive |
| Urambie | Vrn1-WT | WT | Insensitive |

Figure 1 The concentration response curve for Gibberellic acid applied to Spartacus CL with one spray application at the three leaf stage (GS13) and (GS33) with five different concentrations on a logarithmic scale. No significant promotion of flowering with high variability, where error bars are representative of one standard error of the mean of the four replicates.

Figure 2 The concentration response curve for 6-benzyladenine applied to Urambie with one spray application at the three leaf stage (GS13) with five different concentrations on a logarithmic scale. High variability in time to flowering was observed with some concentrations. Error bars are representative of one standard error of the mean of the four replicates for each concentration.

Table 2 The analysis by ANOVA of Experiment 3 when Urambie is excluded from the analysis for the thermal time to GS31 and flowering. Significance of variety, vernalisation and PGR are shown as well as their interactions. Contrasts to the control are displayed, demonstrating the difference of the PGR compared to the control replicate.

|  | **Long days** | |
| --- | --- | --- |
|  | *Thermal time to GS31* | *Thermal time to flower* |
| Variety | <.001 | <.001 |
| Vernalisation | <.001 | 0.004 |
| PGR | <.001 | <.001 |
| CONTROL vs 6-BA | 0.31 | 0.39 |
| CONTROL vs GA | 0.69 | 0.89 |
| CONTROL vs TRE | <.001 | <.001 |
| Variety.PGR | <.001 | 0.45 |
| CONTROL vs 6-BA | 0.31 | 0.61 |
| CONTROL vs GA | 0.8 | 0.35 |
| CONTROL vs TRE | <.001 | 0.47 |
| Vernalisation.PGR | 0.09 | 0.07 |
| Variety.Vernalisation.PGR | 0.09 | 0.006 |

Table 3 The analysis by ANOVA of Experiment 3. Short day and long day analyses were done separately with height (cm) being the measure for comparison at GS31 and flowering. Significance of variety, vernalisation and PGR are shown as well as their interactions. Contrasts to the control are displayed, demonstrating the difference of the PGR compared to the control replicate.

|  | **Short days** | | **Long days** | |
| --- | --- | --- | --- | --- |
|  | *Height at GS31* | *Height at flowering* | *Height at GS31* | *Height at flowering* |
| Variety | <.001 | <.001 | <.001 | <.001 |
| Vernalisation | <.001 | 0.94 | 0.36 | 0.009 |
| PGR | <.001 | <.001 | 0.038 | <.001 |
| CONTROL vs 6-BA | 0.42 | 0.84 | 0.64 | 0.85 |
| CONTROL vs GA | 0.11 | 0.56 | 0.66 | 0.5 |
| CONTROL vs TRE | <.001 | <.001 | 0.023 | <.001 |
| Variety.PGR | <.001 | 0.46 | 0.18 | <.001 |
| CONTROL vs 6-BA | 0.39 | 0.85 | 0.016 | 0.59 |
| CONTROL vs GA | 0.9 | 0.56 | 0.26 | 0.21 |
| CONTROL vs TRE | <.001 | 0.3 | 0.39 | 0.002 |
| Vernalisation.PGR | 0.16 | 0.38 | 0.13 | 0.60 |
| Variety.Vernalisation.PGR | 0.024 | 0.23 | 0.6 | 0.58 |
